# Bilateral clavipectoral fascial plane block in single-stage surgery for bilateral midshaft clavicle fractures

**DOI:** 10.1186/s40981-023-00612-0

**Published:** 2023-04-22

**Authors:** Yoshie Noji, Satoki Inoue, Kazuhiro Watanabe

**Affiliations:** 1 Department of Anesthesiology, Aidu Chuo Hospital, 1-1, Tsuruga-Machi, Aizuwakamatsu, Fukushima 965-8611 Japan; 2 Department of Anesthesiology, Fukushima Medical University, Fukushima, Japan

To the editor,

Clavipectoral fascial plane block (CPB) is used to treat midshaft clavicle fractures [1]. Most clavicle fractures are unilateral, while bilateral fractures are uncommon, and single-stage surgery is also uncommon [2]. We present our experience with bilateral CPB in the treatment of bilateral midshaft clavicle fractures.

A 54-year-old Japanese man (body weight, 64 kg; height, 171 cm) presented with a history of right pneumothorax and reflux esophagitis. He was injured when a tractor overturned and knocked him sideways, resulting in bilateral midshaft clavicle fractures. Pneumothorax developed 2 days after the injury. The pneumothorax improved, and the patient was scheduled for open osteosynthesis of single-stage bilateral midshaft clavicle fracture 9 days after the injury. Bilateral ultrasound-guided CPB was performed after the induction of general anesthesia with positive pressure ventilation. During CPB, an ultrasound probe was placed on the anterior surface of the clavicle. The ultrasound landmarks are shown in Fig. 1. The needle was inserted from a caudal to cranial direction, targeting the clavipectoral fascia and periosteum. When performing left-sided CPB, we injected 10 mL of 0.375% ropivacaine at both the medial and lateral sides of the clavicle fracture. Right-sided CPB was then performed in the same manner. After CPB, the patient was placed in the beach chair position, and surgery was performed. During surgery, desflurane and remifentanil were maintained at 0.1 μg/kg/min without causing significant circulatory changes. Flurbiprofen axetil 50 mg and acetaminophen 1000 mg were administered before surgery ended. The duration of surgery was 210 min, and the duration of anesthesia was 304 min. Figure 2 shows preoperative and postoperative chest radiographs. The patient’s respiratory status was stable after extubation. The postoperative numerical rating scale of pain intensity was 2/10. Additional analgesics were administered at 8 and 19 h after CPB. From postoperative day 1, the patient was able to manage his pain by taking regular loxoprofen.

**Fig. 1:**
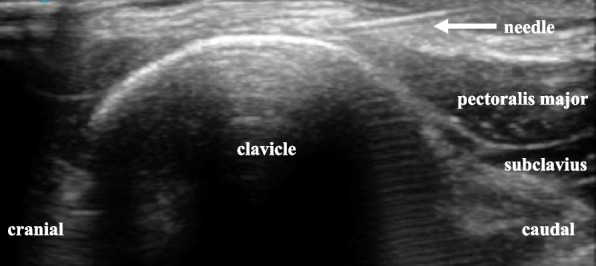
Ultrasound image of clavipectoral fascial plane block

**Fig. 2:**
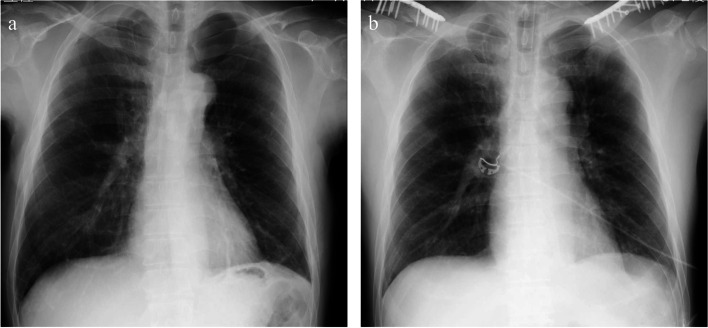
**a** Chest radiograph image was taken 2 days after being injured, revealing bilateral midshaft clavicle fractures and left pneumothorax. **b** Chest radiograph image taken immediately after surgery, showing bilateral repaired clavicle fractures

CPB is a technique to selectively block nerve endings in the clavicle [3]. Interscalene brachial plexus blocks and cervical plexus blocks can be combined for clavicle fractures [1]; however, these approaches also result in phrenic nerve paralysis. CPB selectively blocks clavicle nerve endings and is not associated with phrenic nerve paralysis [1]. In the setting of bilateral midshaft clavicle fractures, such as in this case, CPB is considered a safe analgesic method with few complications. It may also be useful in clavicle fracture surgery for patients with a poor respiratory status or phrenic nerve paralysis on the uninjured side. It has been reported that single-stage awake surgery can be performed for bilateral midshaft clavicle fractures [4]. In this report, we used a combination of general anesthesia and bilateral CPB. We were concerned about the possibility of prolonged surgery and the Bezold-Jarisch reflex caused by the combination of the beach chair position and sympathetic nervous system overstimulation [5]. After consultation with the patient and the surgeon, we decided to use a combination of general anesthesia and bilateral CPB.

We consider that CPB could be useful in single-stage surgery for bilateral midshaft clavicle fractures.

## Data Availability

Not applicable.

## Abbreviation

CPB: Clavipectoral fascial plane block
